# Responsible data sharing in international health research: a systematic review of principles and norms

**DOI:** 10.1186/s12910-019-0359-9

**Published:** 2019-03-28

**Authors:** Shona Kalkman, Menno Mostert, Christoph Gerlinger, Johannes J. M. van Delden, Ghislaine J. M. W. van Thiel

**Affiliations:** 1 Department of Medical Humanities, Julius Center for Health Sciences and Primary Care, University Medical Center Utrecht, Utrecht University, Universiteitsweg 100, 3584 CG Utrecht, the Netherlands; 2 Statistics and Data Insights, Bayer AG, Berlin, Germany; 3 Clinic for Gynecology, Obstetrics and Reproductive Medicine, Saarland University Medical Center, Homburg, Saarland Germany

**Keywords:** Big data, Data sharing, Secondary use, Research ethics, Ethical governance

## Abstract

**Background:**

Large-scale linkage of international clinical datasets could lead to unique insights into disease aetiology and facilitate treatment evaluation and drug development. Hereto, multi-stakeholder consortia are currently designing several disease-specific translational research platforms to enable international health data sharing. Despite the recent adoption of the EU General Data Protection Regulation (GDPR), the procedures for how to govern responsible data sharing in such projects are not at all spelled out yet. In search of a first, basic outline of an ethical governance framework, we set out to explore relevant ethical principles and norms.

**Methods:**

We performed a systematic review of literature and ethical guidelines for principles and norms pertaining to data sharing for international health research.

**Results:**

We observed an abundance of principles and norms with considerable convergence at the aggregate level of four overarching themes: societal benefits and value; distribution of risks, benefits and burdens; respect for individuals and groups; and public trust and engagement. However, at the level of principles and norms we identified substantial variation in the phrasing and level of detail, the number and content of norms considered necessary to protect a principle, and the contextual approaches in which principles and norms are used.

**Conclusions:**

While providing some helpful leads for further work on a coherent governance framework for data sharing, the current collection of principles and norms prompts important questions about how to streamline terminology regarding de-identification and how to harmonise the identified principles and norms into a coherent governance framework that promotes data sharing while securing public trust.

## Background

Recently, a number of multi-stakeholder initiatives have been funded to develop data-driven translational research platforms to improve patient outcomes and reduce the societal burden of specific disease areas in the European Union (EU) [1, 2]. The Innovative Medicines Initiative’s (IMI) BigData@Heart is an example of a consortium that is currently designing an international data sharing platform to stimulate drug development and personalised medicine for cardiovascular disease. To ensure responsible use of data in BigData@Heart as well as similar research projects, good governance of data sharing and data access is critical [1].

So far, no blueprint of a broadly accepted governance framework exists. The recently adopted General Data Protection Regulation (GDPR) (Regulation (EU) 2016/679) will not be able to provide for the necessary guidance in full, since specific provisions for scientific research may still be formulated at the level of national jurisdictions within the EU [3]. Moreover, compliance with the law does not always guarantee that data is used in morally acceptable ways, or that public trust is secured [4]. The evolving landscape of big health data raises new questions about both familiar ethical concepts (such as privacy, confidentiality and informed consent), as well as novel ones. These developments indicate that innovative and adaptable governance models are highly needed to establish a practice of truly responsible data sharing.

To identify what elements are considered inherent to a governance structure for responsible data sharing within (consortium-wide) platforms for international health research, we reviewed frameworks for data sharing as described in the academic literature and in ethical guidelines. This study was driven by the question: What are the ethically relevant principles and norms so far developed by (international) working groups or professional organisations with respect to international data sharing in health research?

## Methods

### Search and selection

We performed a systematic review of principles and norms for responsible health data sharing as identified from the academic, peer-reviewed literature. In addition, we reviewed the principles and norms as developed in a selection of relevant ethical guidelines.

#### Search

Relevant literature was identified through a systematic search in three databases for scientific, peer-reviewed literature, covering the PubMed, EMBASE and Scopus databases (See Appendix 1 for a breakdown of search terms). Search strings were adjusted to the type of database to restrict superfluous results to a minimum (See Appendix 2). Google Scholar was searched for additional sources, including grey literature. Relevant guidelines and policy documents on data sharing in international health research were identified with help from six academic and industry partners from the IMI BigData@Heart consortium with expertise in health law (*n* = 2), regulatory science (*n* = 2) and research ethics (*n* = 2). Experts were asked to list the ethical guidelines they found most relevant to policy and practice.

#### Selection

For inclusion, publications were required to present a coherent set of principles and/or norms that could potentially function as or at least be construed as part of a model or framework for ethically responsible data sharing (Table 1). Here, we understand “principles” to constitute propositions that together serve as the foundation for a system of governing norms. We use “norms” to refer to standards for ethically responsible behaviour or actions. We depart from the assumption that principles inform norms, and that no governance framework can go without these elements. Documents were included if norms and principles were discussed along with tangible measures to facilitate implementation into policy and if the content was developed by or in collaboration with (international) working groups or professional organisations active in the field of health data sharing. Since we were also specifically interested in developments over recent years, we limited our eligibility to sources published between 2006 and 6 August 2018. Only sources published in English were eligible.

**Table 1:** Inclusion and exclusion criteria

Inclusion criteria	Exclusion criteria
Publication describes norms and/or principles for sharing of health data	Publication describes only national and/or EU law
Publication describes coherent set of principles and/or norms that could potentially function as or at least be construed as part of a model or framework for responsible data sharing	Publication describes only benefits, imperatives or challenges for health data sharing or IT infrastructures for Big Data research
Publication discusses norms and principles along with tangible measures to facilitate implementation in policy	Publication not of relevance to the European context
Publication preferably issued by or in collaboration with (international) working groups or professional organisations active in the field of health data sharing	Publication not written in English
Publication published between 2006 and 6 August 2018.	

National and EU laws were excluded from this study because we were primarily interested in elements of a governance framework that provides comprehensive moral guidance, not only enforces legal compliance. Even though the law does require the implementation of a number of organisational and technical measures, what a governance framework exactly looks like is ultimately to be developed in practice [5]. Publications that were limited to a discussion of benefits, imperatives or challenges for health data sharing or IT infrastructures for Big Data research were not deemed relevant to the purpose of this review. All sources that were not of relevance to the European context were also excluded (e.g., practice guidelines for low and middle income countries).

### Data extraction and analysis

From all included references and guidelines we extracted the following data: author names, year of publication, organisation or working group, countries the recommendations apply to (EU/US/international), and the status of the recommendation. By ‘status’ we mean whether the recommendation, for example, has a legal basis, is an ethical guideline, comprises lessons learned, or is an academic proposal. Qualitative content analysis was performed for principles and norms by two independent assessors using the Covidence online support tool for systematic reviews and NVivo qualitative data analysis software (QSR International, Version 11).

## Results

### Selection and data extraction

**Fig. 1:**
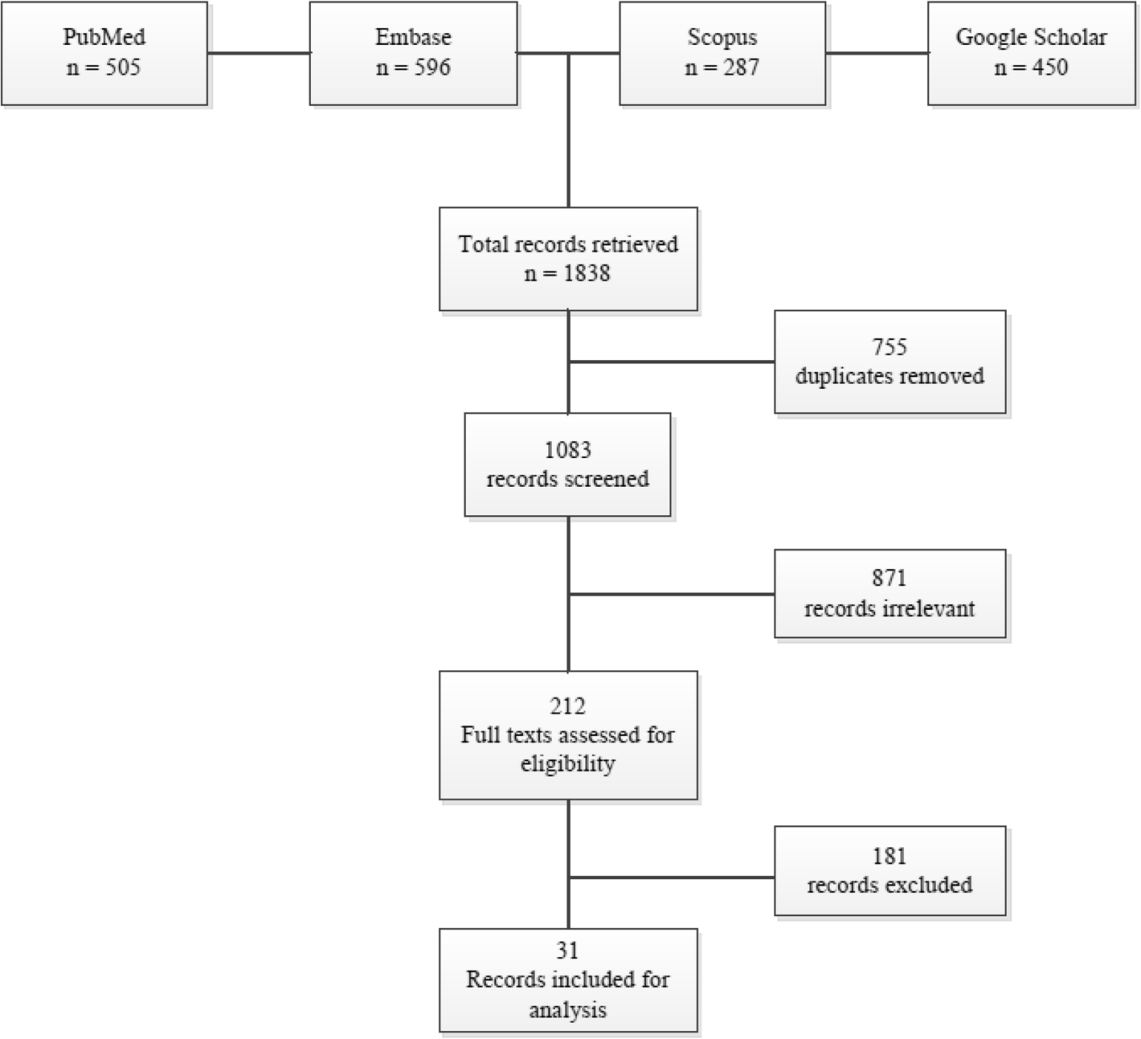
Flow diagram of the selection and inclusion of publications

The literature database searches resulted in a total of 1083 unique records (Fig. 1). Ultimately, we included 31 articles for final review (Table 2). The expert consultation resulted in the inclusion of 10 ethical guidelines by 7 different organisations or working groups (Table 3). The selected guidelines were published between 2007 and 2017. Identified principles and norms were grouped in themes as a means to structure the research findings. Descriptive themes were established through an iterative method and with consensus of all study authors.

**Table 2:** Academic publications included for review

No.	Authors (year)	Organisation, Working group or Institution	Country	Status
1	Board of Directors of the American College of Medical Genetics and Genomics, 2017 [44]	American College of Medical Genetics and Genomics (ACMG)	USA	Policy statement
2	Alfonso, 2017 [7]	International Committee of Medical Journal Editors (ICMJE)	International	Policy statement
3	Allen et al, 2014 [28]	Beacon Community Cooperative Agreement Program	USA	Lessons learned
4	Andrew et al, 2016 [38]	Research collaboration to link data from the Australian Stroke Clinical Registry (AuSCR), the National Death Index, and state held hospital data	Australia	Process evaluation
5	Antman et al, 2015 [25]	American Heart Association Data Summit 2015	USA	Summit report
6	Auffray et al, 2016 [13]	Expert workshop organized by the European Commission (EC)	EU	Expert workshop
7	Baker et al, 2016 [15]	Fair Information Practices Principles (FIPPs) by the Department of Health and Human Services (USA)	USA	
8	Banzi et al, 2014 [41]	Discussion of the European Medicines Agency (EMA) draft policy on publication and access to clinical trial data	EU	Commentary
9	Bredenoord et al, 2015 [29]	International Stem Cell Forum Ethics Working Party	International	Policy statement
10	Chan et al, 2016 [21]	UK National Data Guardian for Health and Care’s Review of Data Security, Consent and Opt-Outs	UK	
11	Chokshi et al, 2006 [34]	MalariaGEN (Malaria Genomic Epidemiology Network)	International	Process evaluation
12	Deverka et al, 2017 [27]	Meet-up of representatives of a range of stakeholders, including healthcare systems, clinical laboratories, technology companies, academia, government, nongovernmental organizations, and patient and community advocacy groups	International	Policy recommendations
13	Dove et al, 2013 [24]	Safe Harbor Framework for International Ethics Equivalency	International	Policy framework
14	Duchange et al, 2014 [36]	EU LeukoTreat program	EU	Process evaluation
15	Dyke & Hubbard, 2011 [11]	Wellcome Trust Sanger, Human Genome Project	International	Process evaluation
16	Dyke et al, 2016 [40]	Global Alliance for Genomics and Health	International	Guidance recommendations
17	Floridi et al, 2018 [33]	European Medical Information Framework (EMIF) project	EU	Process evaluation
18	Knoppers & Thorogood, 2017 [6]	Global Alliance for Genomics and Health	International	Governance framework
19	Knoppers, 2014 [23]	Global Alliance for Genomics and Health	International	Governance framework
20	Knoppers et al, 2011 [26]	Public Population Project in Genomics (P3G), the European Network for Genetic and Genomic Epidemiology (ENGAGE) and the Centre for Health, Law and Emerging Technologies (HeLEX)	International	Code of Conduct
21	Kostkova et al, 2016 [43]	Stakeholder roundtable debate, set up by University College London (UCL)	UK	Policy recommendations
22	Kuehn, 2014 [8]	Institute of Medicine (IOM)	USA	Policy framework
23	Laurie & Sethi, 2013 [32]	Scottish Health Informatics Programme (SHIP)	UK	Guidance recommendations
24	Lea et al, 2016 [14]	The Farr Institute and use of Data Safe Havens	UK	Process evaluation
25	Mascalzoni et al, 2015 [35]	Stakeholder workshop, considered by the Rare Diseases Connect Patient Ethics Council and the Rare Diseases Connect Patient Advisory Council, which endorsed the Draft Charter as the patient consulting bodies of Rare Diseases Connect	International	International Charter of Principles
26	Paltoo et al, 2014 [46]	National Institutes of Health	USA	Policy statement
27	Prainsack & Buyx, 2013 [45]	King’s College and University College London	UK	Governance model
28	Rodriguez et al, 2009 [20]	International summit, convened by the National Cancer Institute (NCI) of the National Institutes of Health (NIH)	International	Amsterdam Principles
29	Shenkin et al, 2017 [39]	BRAINS (Brain Imaging in Normal Subjects) Expert Working Group		Policy recommendations
30	Sugano et al, 2014 [30]	Regulatory and Ethics Working Group, Global Alliance for Genomics & Health	International	Code of Conduct
31	Tucker et al, 2016 [42]	EFSPI (European Federation of Statisticians in the Pharmaceutical Industry) and PSI (Statisticians in the Pharmaceutical Industry) Data Sharing Working Group	International	Policy recommendations

**Table 3:** Selected ethical guidelines and recommendations

Name of source	Organisation, Year	Status	Scope, Addressed to
International Ethical Guidelines for Health-related Research Involving Humans [17]	Council for International Organizations of Medical Sciences (CIOMS), 2016	Ethical guideline, applies to activities designed to develop or contribute to generalizable health knowledge	Universal scope, not defined whom it is addressed to
Declaration of Taipei on Ethical Considerations Regarding Health Databases and Biobanks [31]	World Medical Association (WMA), 2016	Ethical guideline, applies to the collection, storage and use of identifiable data and biological material beyond the individual care of patients	Universal scope, primarily addressed to physicians. The WMA encourages others to adopt the principles.
Declaration of Helsinki - Ethical Principles for Medical Research Involving Human Subjects [37]	World Medical Association (WMA), 2013	Ethical guideline, applies to medical research involving human subjects, including research on identifiable human material and data.	Universal scope, primarily addressed to physicians. The WMA encourages others to adopt the principles
Framework for Responsible Sharing of Genomic and Health-Related Data [10]	Global Alliance for Genomics and Health (GA4GH), 2014	A principled and practical framework, applies to the sharing of genomic and health-related data (for biomedical research)	Universal scope, addressed to all entities or individuals using genomic and health-related data
The collection, linking and use of data in biomedical research and health care: ethical issues [16]	The Nuffield Council on Bioethics, 2015	Report that sets out ethical principles and recommendations, related to the design and governance of data initiatives and data use for biological and medical research	United Kingdom / universal, addressed to anyone approaching a data initiative
Joint statement of purpose—vision, principles, and goals [12]	Funders of public health research, 2011	Joint statement of funders, applies to sharing research data to improve public health	Universal, addressed to funders and the research community
Principles and Guidelines for Access to Research Data from Public Funding [18]	Organisation for Economic Co-operation and Development (OECD), 2007	A legally non-binding recommendation, often referred to as soft law. Applies to research data that are gathered using public funds for the purposes of producing publicly accessible knowledge	Primarily addressed to OECD Member Countries and intended to assist all actors involved when trying to improve the international sharing of, and access to, research data
Recommendation of the Council on Human Biobanks and Genetic Research Databases [19]	Organisation for Economic Co-operation and Development (OECD), 2009	A legally non-binding recommendation, often referred to as soft law. Provides guidance for the establishment, governance, management, operation, access, use and discontinuation of human biobanks and genetic research databases	OECD Countries, to be applied as broadly as possible, in particular to aid policy makers and practitioners who are establishing new human biobanks and genetic research databases. Can also usefully be applied to existing biobanks and databases
Recommendation of the Council on Health Data Governance [22]	Organisation for Economic Co-operation and Development (OECD), 2017	A legally non-binding recommendation, often referred to as soft law. Applies to the access to, and the processing of, personal health data for health-related public interest purposes	OECD member countries and all levels of government, encourages non-governmental organisations to follow this recommendation
Principles for Responsible Clinical Trial Data Sharing: Our Commitment to Patients and Researchers [9]	The European Federation of Pharmaceutical Industries and Associations (EFPIA) and the Pharmaceutical Research and Manufacturers of America (PhRMA), 2013	Joint policy of organisations representing pharmaceutical industries	Members of EFPIA and PhRMA

### Themes, principles and norms

Following data extraction from all sources, the identified principles (and the respective norms promoting those principles) could be grouped among four overarching themes: (1) *Societal benefits and value*; (2) *distribution of risks, benefits and burdens*; (3) *respect for individuals and groups*; and (4) *public trust and engagement* (Table 4).

**Table 4:** Themes and principles

Main themes	Norms and principles
*Societal benefits and value*	Accessibility [7, 10, 15, 19, 23, 25, 26, 30, 32, 34] Data quality [10, 15, 18–20, 23, 24, 29, 30, 35] Sustainability [10, 18, 19, 23, 25, 29, 30] Scientific progress/value [10, 12, 17, 19] Promote health and well-being [9, 10, 37] Interoperability [18, 19, 27] Scientific validity [25, 29, 35] Societal benefit [22, 26, 31] Duty to share [7, 8] Collaboration and capacity building [17, 22] Health-related public interest [22] Improved clinical care [8] Enhance healthcare decision-making [8] Social value [17] Individual benefit [22] Improve public health [12] Efficiency [18]
*Distribution of risks, benefits and burdens*	Benefit sharing [19, 23, 29, 30] Reciprocity [10, 23, 30, 35] Risk-benefit evaluation [13, 14, 16, 32] Equity and fairness [10, 12, 17, 33] Protection of intellectual property [18, 34, 35] Attribution [23, 30, 34] Proportionality [24, 29, 30] Ownership [21, 38] Recognition and attribution [10]
*Respect for individuals and groups*	Respect/protect privacy [11, 12, 15, 19, 22–25, 29–32, 35, 37, 38, 40–42, 44] Protect confidentiality [7, 11, 19, 21, 23, 29–31, 37, 42] Ensure data security [10, 18, 19, 21, 23, 24, 30, 39, 44] Respect individuals [10, 16, 31, 33, 37] Respect individual rights [16, 17, 19, 37] Individual autonomy [11, 31, 35, 37] Respect dignity of individuals [31, 37] Respect (the dignity of) communities [10, 12] Prevent discrimination [19, 31] Legal compliance [24, 25] Protect life, health and well-being [37] Respect families [10] Respect welfare of individuals [17]
*Public trust and engagement*	Transparency [10, 15, 18, 19, 22, 23, 26, 29–31, 33, 43, 44] Accountability [10, 15–19, 21–25, 29–32] Engagement / participation [10, 15–17, 19, 22–25, 27, 31, 32] Maintain public trust [15, 22–27, 31, 32] Maintain integrity [15, 23, 24, 30, 31] Responsibility [21, 23, 25, 29, 39, 42] Professionalism [10, 18, 19, 22] Health democracy [29] Solidarity [45]

#### Societal benefits and value

In most sources, data sharing activities were required to be governed by principles that overall maximise health benefits or wellbeing (both public and individual) and that serve ends of social value. To realise the potential benefits, sources underpin the importance of the *quality* and *comprehensiveness of the data* to be shared, and the *scientific validity* and *social value* of the study protocols submitted by researchers in order to use the data. Once quality and validity have been established, many sources demand a data sharing infrastructure that is *accessible*, enables *efficient* use, is highly *interoperable* and *sustainable* for the future (See Table 4).

In terms of how to bring the principles into practice, sources rely on a wide range of norms, rules and recommendations. First, sources deduce from the potential benefits that there in fact exists a *duty to share* data for scientific research, or a *right to science* [6]. The Institute of Medicine (IOM), the International Committee of Medical Journal Editors and the European Federation of Pharmaceutical Industries and Associations (EFPIA) have come forward with statements about researchers’ and companies’ duty to share their clinical trial data [7–9]. To effectuate *the duty to share*, sources state that awareness about the benefits of data sharing should be raised among stakeholders, and that collaborative partnerships and data sharing practices should be promoted [10]. Other recommendations include devoting efforts and resources to alleviate disincentives for data sharing, such as publication moratoria [11]. The sharing of well-managed datasets and commitments to *disseminate* the results generated from the data (mostly through reports and supporting scientific publications) are considered an equally important element of maximising benefits of data sharing [12–14].

Continuous efforts are considered necessary to improve and maintain *data quality and reproducibility* [15, 16]. Demands with respect to data management and curation include cooperatively developing and implementing quality standards or quality threshold metrics that are submitted to continuous renewal and improvement [17–20]. Sources emphasise the need for data control, compliance with quality standards and feedback mechanisms [10, 18] at every stage of data processing [19]. The use of central repositories is recommended for deposition of data [20]. To maximise *scientific and social value*, data access requests will need to be submitted by qualified researchers who are able to justify the research purposes [21–24], and attest to the use of rigorous scientific methods [9, 25, 26]. Those providing access for secondary use should in turn secure comprehensiveness of the data [20].

*Accessibility* of the data is considered a shared responsibility of researchers, sponsors, research ethics committees and other stakeholders. These actors should work together to (deliver reasonable efforts to) maximise accessibility, and encourage each other to do so too [10, 12, 16, 17, 19, 22]. Accessibility is further enhanced through harmonisation of data access conditions and procedures [13, 27], and by communicating these to stakeholders [10, 18, 28]. One source speaks of the need to establish a “healthy ecosystem” that relies on stakeholder-informed principles and policies that ensure that the needs and concerns of key stakeholders are addressed across different data initiatives [27]. In such an ecosystem there is less emphasis on uniformity of approaches given that some initiatives already have their own governance structures for data sharing in place. Stakeholder-specific incentives to share data and low-cost access to the international research community are ways to increase accessibility [14, 26]. Many sources consider the development of strategies, processes and/or systems that help secure long-term accessibility and *sustainability* of the organisation of great importance (e.g., through funding) [10, 18, 19, 23–25, 29, 30]. It should be made clear how the data will be dealt with in the event of discontinuation of the data holder [17, 19], or a change of ownership [31]. Uniform policy is required with respect to the duration of storage [19], and the disposal and destruction of data [31].

*Interoperability* is enhanced by cataloguing data in a consistent manner [14], according to internationally accepted standards and norms [10, 18, 19], by incorporating standardised design elements that provide for compatibility [19], and through harmonisation of regulatory frameworks for data sharing in Europe [13]. Documentation of data quality and origin should be readily available, verifiable [19], accurate, unbiased and proportionate [10]. For those who have been granted access to data, validation exercises should be allowed whenever possible [18].

#### Distribution of risks, benefits and burdens

Many sources require that the burdens and benefits of data sharing are fairly allocated. In other words, data sharing efforts should adhere to principles of distributive justice (See Table 4). Benefits to individuals and society should be maximised and harms should be minimised and thus should also be *proportional* [24, 29, 30]. *Benefit sharing* and *reciprocity* is distinguished between participants and researchers, as well as between researchers, secondary users, communities and funders [12, 22, 32]. One source states that it should be assured that benefits are shared “as broadly as possible” [19], especially when data is collected from vulnerable communities [31]. *Equitable access* is ensured by transparency rules, fair access fees and a balance between the needs of data holders, secondary users and the communities who expect health benefits to arise from the research [19, 22, 26]. Commercial interest is generally not considered a reason to restrict access to data. One source states that the criterion of commercial versus non-commercial research is actually not very helpful, since research carried out for commercial reasons or by commercial companies can in fact be very beneficial to society [33]. Instead, access should be based on balanced arrangements between public and private parties [18] and whether or not these parties are “bona fide”, meaning that their research serves the ultimate goal to discover “new knowledge intended for the general interest in health and to be made publicly available without undue delay” [33].

Sources also emphasise the need for establishing adequate systems for *recognition, ownership* and *attribution*, that are designed in such a way that due credit and acknowledgment is given to all who contributed to the results. To these principles between data holders and secondary users, sources call upon the application of intellectual property (IP) laws to data access arrangements [18, 31, 34]. According to some sources, policy should make sure to cover benefit sharing and IP issues as transparently as possible, and for it to be communicated appropriately [19, 31]. Researchers are required to report back to the relevant data holders a list of publications and patent issues arising from the database’s resources [10, 19, 35]. However, others sources point out that exclusive ownership runs counter to the goals of data sharing initiatives [27, 33]. This would hold for individuals whose data is being shared but also for other actors involved in data sharing activities. A solution recommended includes inserting a “perpetuity” clause as a condition for making data available in a data sharing platform [33]. The clause would only allow withdrawal of the data in case the grounds for making them available have changed.

#### Respect for individuals and groups

Respect for individuals and groups is represented by an abundance of identified principles, norms, rules and recommendations (See Table 4). From the principle of *respect for autonomy* it follows that the purposes to which data is shared should be consistent with the (scope of the original) informed consent [9, 17, 19, 25, 35]. Some sources differentiate between ‘specific informed consent’ and ‘broad informed consent’ or ‘dynamic informed consent’ for a range of future data uses [16, 17, 19, 31]. When future use is specified at the time of data collection, or the data are collected for a given research project, specific informed consent from individuals is required [17, 31]. When this is not the case, some sources permit the conditional use of broad consent models [16, 17, 19, 22, 31]. Valid broad informed consent relies on certain (additional) safeguards, such as a proper governance framework and the provision of sufficient information to participants [16, 17, 19, 31]. During and/or after the informed consent process, sources state that participants should be informed about the topics as listed in Table 5. Clear and easy-to-use processes should remove barriers for participants to withdraw their consent for the use of their data at any time [17, 19, 22, 31]. *Rights* that are considered relevant for participants are listed in Table 5. Furthermore, policies and procedures are recommended for when and how to re-contact participants [16, 17, 19, 31, 36], in particular with respect to the return of unsolicited findings, and how participants can request access to their data [15, 19].

**Table 5:** Informing and enabling participants and the public

Potential participants need to be informed about:	
–the type of research being carried out, the activities of health databases and/or the research results [10, 19, 31];	
–the legal basis and objectives of the data processing by third parties [22];	
–how consent can be withdrawn, as well as the implications of and limits to withdrawal [17, 19, 31];	
–whether the participants retain any rights over the data [19];	
–whether return of individual-level findings derived from analysis of the data is foreseen and the right to opt-out from receiving such information [17, 19];	
–how the data and the confidentiality of these data will be protected [17, 19];	
–the limits to anonymity and confidentiality of data [10, 16, 17, 19];	
–the exceptional circumstances and conditions under which researchers may access data that is not coded or anonymous [19];	
–the potential adverse consequences of breaches of confidentiality [17];	
–information about an actual significant data breach or misuse of data [22];	
–significant modifications to databases’ policies, protocols and procedures [19];	
–entering into commercial collaborations or commercialisation of research resources [19].	
Enable participants to exercise the following rights:	
–the right to withdraw consent [17, 19, 22, 31];	
–the right to choose whether (and how) individual-level findings will be returned [17, 19];	
–the right to request for information about their data and its use [31];	
–the right to request for corrections of omissions in data [31];	
–the choice to opt-out of being re-contacted for research purposes [17].	
Related to data sharing, public information should include the following items:	
–the terms, procedures, policies and/or governance frameworks for data access or sharing [10, 16, 18, 19, 22];	
–for what purposes and ways in which data may be shared [10, 16, 22];	
–a summary of (approved) data transfers [10], including a list of categories of approved data recipients [22];	
–the legal bases for sharing data [22];	
–a catalogue of the resources accessible for research purposes [19];	
–the duration of data storage [10];	
–a specification of conditions attached to the use of the data [18];	
–a summary of research results [19];	
–commercial involvement and propriety claims [10];	
–processes of withdrawal from data sharing [10];	
–contact information and answers to frequently asked questions [19];	
–procedures for handling complaints [31];	
–the purpose, background, funding, scope, uncertainties and risks, scientific rationale of the initiative or database and its funding [19];	
–the disclosure of any conflict of interest involving personnel [19].	

If informed consent for data access cannot reasonably be obtained (“impossible” or “impracticable”), waivers of informed consent may potentially be issued [17, 19, 31, 37, 38]. Some of the sources state that waivers of informed consent for data (re-)use should be issued after approval of a research ethics committee (REC) only, and “in accordance with applicable law” and “ethical principles” [19, 39]. The Declaration of Taipei restricts waivers to the event of a “clearly identified, serious and immediate threat (...) to protect the health of the population” [31], while the Council for International Organizations of Medical Sciences (CIOMS) guidelines demand that the study has important social value and poses “no more than minimal risks” [17]. An alternative is to have RECs allow the conditional use of an ‘informed opt-out’ procedure [17]. Even in cases where no express consent has been given, however, individuals should be able to express preferences regarding the use of their data—at least to the extent practicable [22].

Norms that help protect *privacy* and *confidentiality* include the establishment and periodical updating of security measures, protocols and other protective safeguards [15, 16, 18, 19, 21, 22, 31, 40], which are proportionate to the use and nature of the data [10, 37]. Substantial support was observed among sources for the requirement to only store and share data that is de-identified (anonymised or coded) [19, 21, 41, 42]. At the same time, the limits of anonymity and confidentiality are acknowledged and should be anticipated [17, 19]. One source states that use of anonymised data should generally be avoided because it makes it impossible to add patient-level data and/or to re-contact participants [35]. In all cases, researchers are said to have the obligation to inform individuals that complete confidentiality can never be guaranteed [35]. There is agreement among sources on the rule that the sharing of identifiable data or permission for re-identification should only be allowed for research purposes (unless ordered by law) and after approval “conform applicable procedures” [19, 22]. Terms include access limitation to those with a need-to-know [21], and restrictions on who may have (third party) access to (potentially) identifiable data [15, 17, 19].

*Data security* is further enhanced if technical alternatives for physical transfer of data are explored, such as the use of secure data access centres and remote data access facilities [22, 35]. To prevent unauthorised access or any other misuse, robust infrastructures will need to arrange for identity verification and authentication before access is granted [19, 21, 22]. Infrastructures should also monitor and document any access to identifiable data [19], and implement feedback mechanisms for data security [10]. Policy should include statements about how confidentiality is practically maintained [17], and that users must refrain from any attempt to (re-)identify participants [10, 16]. Essential to secured sharing is education and training of researchers on issues such as data security and privacy compliance [14, 43].

#### Public trust and engagement

Many sources report on principles and norms that relate to maintaining *public trust* and engaging in *public and patient involvement* and/or *participation.* Public trust and engagement constitute a theme that has instrumental value to maximise benefits, promote respect for persons, minimise harms *and* to protects principles of social justice. Nevertheless, we treat public trust and engagement as a separate moral category to illustrate the emphasis that it has been given in the reviewed sources [14, 43]. Key principles reported by the sources that foster public trust and engagement are shown in Table 4.

According to reviewed sources, strategies used by data sharing initiatives should be built upon *trust*, which is gained by being *trustworthy* [27]. Sources emphasise the need to develop formats and mechanisms that enable effective deliberation with relevant stakeholders—including participants, the public, funders and the research community—about important issues of data sharing [10, 13, 16, 17, 19, 25, 28]. More specifically, participation should be increased in the design, governance and review of data initiatives—of which the results should eventually translate into policy [8]. Preferably, a regular process of reviewing and modifying data access policies, protocols and procedures should be in place [18, 19], which pays heed to relevant issues that may change over time (e.g., IT, legal and/or cultural issues) [18]. Other opportunities for patient and public involvement include events and workshops to disseminate research findings, as well as organising lay presentations or panels, steering committees and working groups to give participants a meaningful voice in governance regarding their data [14, 21, 26]. One source explicitly places the participant at the center of the data sharing infrastructure, so that individuals whose data is shared are more meaningfully empowered to make decisions about access and use [27]. Through trusted intermediaries and easy-to-use tools individuals would be able to more easily contribute and control use of their data [26].

The principle of *transparency* can be brought into practice through different mechanisms. First and foremost, transparency needs to exist in all workflow of data sharing activities and transactions (including documentation) [15, 23, 29, 30, 44]. Especially transparency in data sharing transactions is flagged as an essential component of responsible data sharing. The principle is also effectuated through the dissemination of public information about ongoing data sharing activities [43]. Items that are proposed to be included in such public information are listed in Table 5. At the same time, researchers and institutions will need to raise awareness and increase understanding among the public towards the need for data sharing to democratise health research [21, 29, 43].

Special consideration was given to the importance of effective governance systems as a means to promote *integrity, solidarity* and *accountability* in data sharing activities [15–17, 22, 43, 45]. Each international collaborative data research initiative is expected to operate “within an explicit public ethics and governance framework” [16]. The governance structure should clearly outline the *responsibilities* of designated individuals or entities [21], establish measures for accountability (e.g., whether secondary use has met the intended purposes and sanctions for breaches) [21], and install mechanisms for monitoring, audits and general oversight (e.g., good stewardship of stored data) [16, 17, 19, 21, 22]. A more specific recommendation is to establish a governance committee to oversee policy developments [11]. Compliance with existing legal requirements, ethical principles and collaborative agreements is considered paramount [19, 21, 24, 25, 33, 39]. Particularly, investments need to be made in fostering *professionalism*—which involves education and training of professionals and other staff—and communication with participants and the public [14, 19, 43]. *Social accountability* arises from engagement of individuals in society, supported by organisations that communicate to individuals and society about the expectations and failures of data governance [16].

In most sources, review and approval procedures by an independent REC (or comparable review body) play an important part in discussions about responsible data sharing for health research [14, 39, 46]. Some sources state that an REC (or comparable body) must review and approve every study using collected data [17, 19, 31, 39]. Some aspects of REC review have already been discussed in the context of respect for individuals and groups. The full list of items or situations that are considered subject to ethics review and approval can be found in Table 6. Data access should be based on the legitimacy of the research purpose [9], objective and clearly articulated criteria (as recorded in policy documents) [24], and restricted to researchers who have received adequate data security training [14], and who are subject to institutional oversight and effective sanctioning [16, 19, 21]. When access to data is granted, agreements should specify the terms of access [19, 22, 31]. Transactions can be responsibly facilitated through the use of binding data access agreements (DAAs), such as data transfer agreements (DTAs) [7, 29, 32, 36, 42]. Ideally, these DAAs follow a standardised format to regulate access uniformly and consistently. DAAs should include arrangements to promote good practices to enable quality control [18], arrangements for a secure transfer [22], and appropriate and effective means to sanction misuse or non-compliance [22, 27].

**Table 6:** Items subject to ethical review

An REC (or a comparable ethical review body) should review:	
–whether the consent given is sufficient for the planned use [17, 31];	
–for determining when to seek re-consent [19];	
–use of data on the basis of broad consent [19];	
–(the justification of) a waiver of informed consent requirements [17, 19, 31, 37];	
–Usage of data not anticipated in the original informed consent process [19];	
–Re-use in cases where informed consent may not have been obtained previously [19];	
–whether the consent procedure meets the specifications of broad informed consent [17];	
–whether explicit informed consent is required [17];	
–whether an informed opt-out procedure can be used [17];	
–the proposed usage and/or collections, the storage protocol [17];	
–if other measures need to be taken to protect the donor [31];	
–the use of personal identifiers, its necessity and how confidentiality will be protected [17];	
–whether individual counselling is necessary when returning genetic findings [17].	

## Discussion

This systematic review of the academic literature and research ethical guidelines provides a unique overview of principles and norms that are considered inherent to a governance framework for responsible data sharing. Content of 31 international academic publications and ten guidelines was qualitatively analysed. We observed an abundance of principles and norms with considerable convergence at the aggregate level of four overarching themes: societal benefits and value; distribution of risks, benefits and burdens; respect for individuals and groups; and public trust and engagement.

In terms of societal benefits and value, it is considered necessary by some to raise awareness about the duty to share health data, and to secure that only high-quality data is shared for scientifically valid proposals. Systems for data sharing should allow for efficient use, and be highly interoperable and accessible, as well as sustainable for the future. To ensure fair distribution of risks, benefits and burdens, effective mechanisms for benefit sharing will need to be in place. Collective evidence generation requires governance that has systems for recognition, attribution and ownership built in. Respect for individuals and groups covered a range of identified principles and norms, among which the principles to respect privacy and confidentiality were by far the most prominent. There is a growing consensus that absolute anonymity or confidentiality cannot be guaranteed, despite the common requirement to de-identify data to protect privacy. Moreover, because of the nature of data sharing activities, it is acknowledged that alternatives will need to be devised for traditional, specific informed consent. What is more, it is recommended in most of the sources that an ethics committee (or a comparable body) reviews and approves data access requests. Lastly, public trust is crucial to responsible data sharing. In this relation, accountability, transparency, integrity and professionalism are key principles. Continued stakeholder engagement, from study design to the dissemination of research findings, can and should be facilitated using different methods.

At the level of principles and norms we observed substantial variation in: (1) the phrasing and level of detail of principles and norms, (2) the number and content of norms considered necessary to protect a principle, and (3) the contextual approaches in which principles and norms are used. An example of point (1) is that some sources reported only in very general terms on relevant principles (e.g., “data sharing should be transparent” or “access should be ensured”), while others provided more detailed descriptions (e.g., “the public should be continuously updated about ongoing data sharing activities” or “ensure low data access fees”). Point (2) is exemplified by the diversity of norms related to informed consent and exemptions from (specific) consent requirements. Only some of the sources explicitly allow the conditional use of broad informed consent models or opt-out procedures. With respect to point (3), whereas one source would discourage the use of anonymised data other sources would actually demand complete de-identification. While the identified principles and norms provide helpful guidance on an impressive range of items, these three points indicate that the current collection of principles and norms still requires further work on how to exactly incorporate principles and norms into a coherent yet adaptable governance framework for health data sharing. Although different collaborative partnerships have already undertaken steps towards the latter [47, 48], we stress the need for continued efforts to further develop and implement such a governance framework for international data sharing projects [5].

A particular issue of importance we wish to address here is that our analysis also points to a confusion in the meaning of terms used to describe the degree and type of data de-identification. This could affect both the sharing, security and confidentiality of that data. Our findings support the notion of what Phillips and Knoppers have labelled a “Babel-like lexicon for de-identified data” [49]. While funders and research organisations push towards increased data sharing, there are legal duties that require ‘de-identification’ to protect privacy. We found that one fairly undisputed recommendation is to inform participants about the limits of anonymity and confidentiality. However, the extent to which principles and norms apply to data with varying degrees of de-identification remains largely unclear. The GDPR provides no guidance for sharing of de-identified data because it only applies to the use of personal data. Our findings lead us to suspect that reviewed authors define the terms ‘anonymisation’ and ‘pseudonymisation’ in different ways. For example, the terms ‘anonymous’, ‘anonymised’ and ‘de-identified’ seemed to be used interchangeably. Yet there is a moral difference between collecting data without direct identifiers and removing those direct identifiers later on [49]. We recommend that a governance framework (1) clearly defines the terms it uses and 2) goes beyond simply acknowledging the limits of anonymity and/or requiring de-identification at all costs (at the expense of data quality). The key to resolving limitations in anonymity lies in the explicit connection with public trust.

The themes we have identified share considerable similarities with the moral considerations of a framework for public health ethics [50]. This suggests that the ethics of international data sharing is probably best captured by moral duties that arise from the interactions and relationships between health care professionals, various public and private actors and the public. We hasten to mention that our thematic categorisation is not intended as a new governance framework in itself. Rather, our thematisation helps to identify common grounds and to structure various principles and norms in such a way that the basic structure of a governance framework becomes visible. We acknowledge that certain principles could be categorised as belonging to more than one theme, and norms and recommendations as serving more than one principle. With respect to our search strategy, the terms ‘principles’ and ‘norms’ may have been used differently in the literature and guidelines than the way in which we defined them. We could have missed sources that have not used these terms but in fact do refer to notions that fit our description of principles and norms. Nonetheless, we believe that the reviewed sources are informative to the establishment of a governance framework for data sharing.

This review was also limited to expert-selected guidelines and a selection of peer-reviewed literature on the topic of data sharing for health research. We are aware that our findings, particularly the body of sources identified by experts, cannot make any claims to comprehensiveness. A plethora of policy statements on data access and data sharing exists at the level of governmental bodies, industry [51], regulatory agencies (such as the European Medicines Agency [52]), and public and private institutions. A recent publication analysed data sharing guidelines to explore why data is not shared more broadly in the medical sciences [53]. Blassime and colleagues found that three themes were referred to much more frequently than others, namely: data subjects’ autonomy and privacy, and data quality and curation, though these themes were not given the same appreciation by the different organisations. At the same time, the authors observed substantial fragmentation in the landscape of data sharing policies. The findings of Blassime and colleagues [53] support the results of our review in the sense that central themes (or ‘principles’) were uncovered but their contextual use varied and thus leads to under- and sometimes oversharing of health data.

## Conclusions

In this study we aimed to capture what principles and norms have been formulated by (international) collaborative working groups and organisations with respect to responsible data sharing in international health research. We believe that the four themes (societal benefits and value; distribution of risks, benefits and burdens; respect for individuals and groups; and public trust and engagement) under which relevant principles and norms can be grouped, reflect what authors, organisations and working groups consider aspects of importance to governing data sharing activities in a responsible manner. These insights provide helpful leads for further work on conceptualising a harmonised governance framework for data sharing in health research. At the same time, our findings indicate substantial variation in: (1) the phrasing and level of detail of principles and norms, (2) the number and content of norms considered necessary to protect a principle, and (3) the contextual approaches in which principles and norms are used. Key questions, in particular how to streamline terminology regarding data de-identification and how to harmonise the identified principles and norms into a coherent governance framework, will have to be part of the research agenda.

## Appendix 1

**Table 7:** Breakdown of search terms

“Data-sharing”	AND	Guideline*	AND	International*
“Data-linkage”		Guidance		Supranational*
“Data-intensive”		Code*		Normative
“Big Data”		Recommendation*		Moral*
		Governance		Ethic*
		Declaration		Legal*
		Regulatory		
		Regulation*		
		Framework		

## Appendix 2

**Table 8:** Search queries (performed August 6, 2018)

Database	Search string	Returned
PubMed	(((((((international*[Title/Abstract]) OR supranational*[Title/Abstract]) OR ethic*[Title/Abstract]) OR moral*[Title/Abstract]) OR normative [Title/Abstract]) OR legal*[Title/Abstract])) AND (((((((((((Guideline*[Title/Abstract]) OR Guidance [Title/Abstract]) OR Code*[Title/Abstract]) OR Recommendation*[Title/Abstract]) OR Governance [Title/Abstract]) OR Declaration [Title/Abstract]) OR Regulatory [Title/Abstract]) OR Regulation*[Title/Abstract]) OR Framework [Title/Abstract])) AND ((((“big data”[Title/Abstract]) OR “data-sharing”[Title/Abstract]) OR “data-linkage”[Title/Abstract]) OR “data-intensive”[Title/Abstract]))	505
Embase	(‘data sharing’:ab,ti OR ‘big data’:ab,ti OR ‘data linkage’:ab,ti OR ‘data intensive’:ab,ti) AND (‘guideline*’:ab,ti OR ‘guidance’:ab,ti OR ‘code*’:ab,ti OR ‘recommendation*’:ab,ti OR ‘governance’:ab,ti OR ‘declaration’:ab,ti OR ‘regulatory’:ab,ti OR ‘regulation*’:ab,ti OR ‘framework*’:ab,ti) AND (‘international*’:ab,ti OR ‘supranational*’:ab,ti OR ‘normative’:ab,ti OR ‘moral*’:ab,ti OR ‘ethic*’:ab,ti OR ‘legal*’:ab,ti)	596
Google Scholar	allintitle: health OR framework OR governance OR recommendations “data sharing” [limit 2006–2017]	450
Scopus	TITLE-ABS (“data sharing” OR “data linkage” OR “data intensive” OR “big data”) AND TITLE-ABS (guideline* OR guidance OR code* OR recommendation* OR governance OR declaration OR regulation* OR regulatory OR framework*) AND TITLE-ABS (international* OR supranational OR normative OR moral* OR ethic* OR legal*) AND TITLE-ABS (medical OR health) AND TITLE-ABS (research)	287
